# Reduced Levels of miR-145-3p Drive Cell Cycle Progression in Advanced High-Grade Serous Ovarian Cancer

**DOI:** 10.3390/cells13221904

**Published:** 2024-11-18

**Authors:** Eva González-Cantó, Mariana Monteiro, Cristina Aghababyan, Ana Ferrero-Micó, Sergio Navarro-Serna, Maravillas Mellado-López, Sarai Tomás-Pérez, Juan Sandoval, Antoni Llueca, Alejandro Herreros-Pomares, Juan Gilabert-Estellés, Vicente Pérez-García, Josep Marí-Alexandre

**Affiliations:** 1 Research Laboratory in Biomarkers in Reproduction, Obstetrics and Gynecology, Research Foundation of the General University Hospital of Valencia, 46014 Valencia, Spain; evagonzalezcanto@gmail.com (E.G.-C.); dra.kristina.agababyan@gmail.com (C.A.); sarai.altea@gmail.com (S.T.-P.); juangilaeste@yahoo.es (J.G.-E.); 2 Bioinformatics and Genomics Department, Saphetor SA, 1015 Lausanne, Switzerland; marianabraga.m@gmail.com; 3 Department of Obstetrics and Gynecology, General University Hospital of Valencia Consortium, 46014 Valencia, Spain; 4 Research Laboratory of Molecular Mechanisms of Placental Invasion, Centro de Investigación Príncipe Felipe, 46012 Valencia, Spain; aferrero@cipf.es (A.F.-M.); snavarro@cipf.es (S.N.-S.); mmellado@cipf.es (M.M.-L.); 5 Epigenomics Unit, La Fe Health Research Institute, 46026 Valencia, Spain; epigenomica@iislafe.es; 6 Biomarkers and Precision Medicine Unit (UByMP), La Fe Health Research Institute, 46026 Valencia, Spain; 7 Department of Obstetrics and Gynecology, General University Hospital of Castellón, 12004 Castellón de la Plana, Spain; antonillueca@gmail.com; 8 Multidisciplinary Unit of Abdominal Pelvic Oncology Surgery (MUAPOS), General University Hospital of Castellón, 12004 Castellón de la Plana, Spain; 9 Department of Medicine, University Jaume I, 12006 Castellón de la Plana, Spain; 10 Gynaecological Oncology Laboratory, Department of Oncology, KU Leuven, 3000 Leuven, Belgium; alejandro.herrerospomares@kuleuven.be; 11 Department of Pediatrics, Obstetrics and Gynecology, University of Valencia, 46014 Valencia, Spain; 12 Centro de Biología Molecular Severo Ochoa, Consejo Superior de Investigaciones Científicas (CSIC), Universidad Autónoma de Madrid (UAM), 28049 Madrid, Spain; 13 Department of Pathology, General University Hospital of Valencia Consortium, 46014 Valencia, Spain

**Keywords:** high-grade serous ovarian cancer, miRNAs, DNA methylation, miR-145-3p, cell cycle proliferation, cyclin D1-CDK4/6 pathway

## Abstract

High-grade serous ovarian cancer (HGSOC) is the most lethal form of gynecologic cancer, with limited treatment options and a poor prognosis. Epigenetic factors, such as microRNAs (miRNAs) and DNA methylation, play pivotal roles in cancer progression, yet their specific contributions to HGSOC remain insufficiently understood. In this study, we performed comprehensive high-throughput analyses to identify dysregulated miRNAs in HGSOC and investigate their epigenetic regulation. Analysis of tissue samples from advanced-stage HGSOC patients revealed 20 differentially expressed miRNAs, 11 of which were corroborated via RT-qPCR in patient samples and cancer cell lines. Among these, miR-145-3p was consistently downregulated post-neoadjuvant therapy and was able to distinguish tumoural from control tissues. Further investigation confirmed that DNA methylation controls *MIR145* expression. Functional assays showed that overexpression of miR-145-3p significantly reduced cell migration and induced G0/G1 cell cycle arrest by modulating the cyclin D1-CDK4/6 pathway. These findings suggest that miR-145-3p downregulation enhances cell proliferation and motility in HGSOC, implicating its restoration as a potential therapeutic target focused on G1/S phase regulation in the treatment of HGSOC.

## 1. Introduction

Ovarian cancer (OC) constitutes one of the most prevalent and lethal gynecological malignancies worldwide, staggeringly impacting women’s health [1]. Within the context of OC, the most frequent are epithelial tumours, specifically high-grade serous ovarian cancer (HGSOC) (70%), followed by endometrioid carcinomas (10%) and clear cell carcinomas (5%). Unfortunately, over 80% of patients are diagnosed at advanced stages (stage IIIC or IV), decreasing the 5-year overall survival (OS) rate from 92% at early stages to 29% in advanced ones [2]. Metastases, particularly in the omentum, high recurrence rates, and chemoresistance contribute to OC’s poor prognosis [3].

To date, limited therapeutic strategies with moderate success exist for this malignancy. Standard first-line treatment for advanced-stage OC includes cytoreductive surgery followed by platinum- and taxane-based chemotherapy [4]. Recent therapeutic advances, such as poly(ADP-ribose) polymerase inhibitors (PARPis), particularly benefited patients with homologous recombination deficiency tumours [5]. In OC, PARPis were initially developed as maintenance therapy in patients with complete or partial response after platinum-based chemotherapy for recurrent disease [6]. Up to now, trials evaluating PARPis as first-line treatment demonstrated significantly improved progression-free survival, although overall survival results are not yet mature for any of the trials [7,8,9,10]. Moreover, despite the increasing recognition of the immune system’s crucial role in OC prognosis, immune checkpoint inhibitor therapies, successful in other solid tumour types, have yet to demonstrate comparable efficacy in OC [11,12].

Epigenetics emerged as a pivotal player in OC pathogenesis. Among the extensively studied epigenetic mechanisms, DNA methylation and non-coding RNAs, particularly microRNAs (miRNAs), garnered attention. Both mechanisms significantly influence gene expression and, consequently, various stages of cancer development, encompassing initiation, promotion, and progression, including metastasis [13]. miRNAs are small non-coding RNAs (19–22 nucleotides) that can act as post-transcriptional regulators of gene expression, inhibiting translation or promoting degradation of their target mRNAs [14]. DNA methylation, on the other hand, involves the covalent addition of a methyl group to cytosine’s 5′ carbon, with hypermethylation at the CpG islands of gene promoters repressing gene expression and hypomethylation leading to overexpression. Interestingly, about 50% of miRNA genes present CpG islands, suggesting their expression may be influenced by DNA methylation.

In the context of OC, previous research identified aberrantly methylated miRNA genes associated with distinct steps of pathogenesis and metastatic spread [15,16], suggesting their potential as therapeutic targets and biomarkers. Notably, epigenetic therapies, particularly demethylating agents as Decitabine and Azacitidine, effective in hematological malignancies, might be explored for potential applications in OC. The clinical advancement of miRNA-based epigenetic therapies further opens avenues for innovative and effective treatments [17,18].

In the pursuit of unraveling the intricate molecular mechanisms underpinning OC, we conducted miRNA sequencing, investigated putative DNA methylation regulation, and performed functional studies to identify differentially expressed miRNAs (DEmiRNAs) involved in ovarian carcinogenesis. This comprehensive exploration seeks to contribute not only to our understanding of OC pathobiology, but also to the development of novel therapeutic strategies with the potential to enhance patient outcomes. Through our investigations, we shed light on the deregulated miRNAs in HGSOC, particularly highlighting the sustained downregulation of miR-145-3p post-neoadjuvant treatment. Functional assays unveiled its significant role in reducing cell migration and inducing G0/G1 cell cycle arrest, implicating the modulation of the cyclin D1-CDK4/6 pathway. These findings position the restoration of miR-145-3p levels as a potential therapeutic target among G1/S regulators in HGSOC treatment strategies, offering promising avenues for further research and clinical intervention. 

## 2. Material and Methods

### 2.1. Patients and Tissue Samples

#### 2.1.1. Study Cohort

The study consists of a retrospective multicentre study. Patients (n = 20) were surgically treated and recruited either at the General University Hospital of Valencia or the General University Hospital of Castellon (Spain) between October 2017 and July 2021.

Inclusion criteria comprehended patients undergoing surgical resection for suspected OC with histological confirmation of HGSOC in the affected ovary and absence of malignancy in the paired control ovary and patients who signed the informed consent. All the tumours displayed criteria of HGSOC, including a serous tumour with a solid component, encompassing papillary, glandular, or cribriform architecture, large atypical nuclei, and high mitotic activity. All the samples displayed immunoreactivity for WT1, CK7, PAX8, and p16 and were negative for NapsinA. Exclusion criteria included non-HGSOC diagnoses (including but not restricted to low-grade serous ovarian cancer or borderline serous histologies) and absence of paired control non-tumoural ovarian tissue (PCOT). Demographic variables (age, body mass index (BMI), and ethnicity) were obtained pre-surgery, and peritoneal carcinomatosis index (PCI) [19] and stage (as per FIGO 2014 staging system [20]) were recorded according to surgical findings.

#### 2.1.2. Ethics Statement

All participants signed the informed consent. The research adhered to the ethical principles of the Declaration of Helsinki and its successive amendments [21]. The study was approved by the Institutional Review Board of our institution (JGE-TAX-2017-01 09282017).

#### 2.1.3. Sample Collection

From each patient, both tumoural and PCOT samples were processed according to the established protocol. Briefly, formaldehyde-free samples were directly brought to the Pathology Service, where experienced pathologists macroscopically dissected fragments of ovarian tissue not required for diagnosis. Adjacent tissue sections were analysed to confirm the presence (tumour group) or absence (PCOT group) of HGSOC. Fresh tissue samples were preserved in RNAlater (Applied Biosystems, Thermo Fisher Scientific; Waltham, MA, USA) and stored at −80 °C until processing.

### 2.2. Cell Lines and Cell Culture

The human OC cell lines Caov-3 (HTB-75) (RRID:CVCL_0201), SK-OV-3 (HTB-77) (RRID:CVCL_0532), and SW-626 (HTB-78) (RRID:CVCL_1725) were purchased from the American Type Culture Collection (ATCC; Manassas, VA, USA). Cells were grown in DMEM medium (ATCC; Manassas, VA, USA), McCoy’s 5A Modified Medium (Gibco, Thermo Fisher Scientific; Waltham, MA, USA), and Leibovitz’s L-15 Medium (Gibco, Thermo Fisher Scientific; Waltham, MA, USA), respectively, supplemented with 10% of FBS (Gibco, Thermo Fisher Scientific; Waltham, MA, USA) and 1% penicillin/streptomycin (Gibco, Thermo Fisher Scientific; Waltham, MA, USA), under 5% CO_2_ and 37 °C. All cell lines were incubated in a 5% CO_2_ humidified atmosphere at 37 °C and culture media was renewed every 48 h. All cell lines tested negative for mycoplasma.

### 2.3. RNA Isolation from Fresh Biopsies and Cell Lines

Total RNA, including the miRNA fraction, was extracted from 25 mg of fresh tissues, and from 10^5^ OC cells using the mirVana miRNA Isolation Kit (Ambion, Thermo Fisher Scientific; Waltham, MA, USA), following manufacturer’s recommendations. Prior to the extraction, fresh tissues were fragmented with a scalpel and homogenised using the Fisherbrand 150 handheld homogeniser (Fisherbrand, Thermo Fisher Scientific; Waltham, MA, USA).

Total RNA from OC-transfected cells was extracted using the TRIzol reagent (Ambion, Thermo Fisher Scientific; Waltham, MA, USA) according to manufacturer’s instructions.

RNA purity was assessed by quantifying the A_260_/A_280_ and A_260_/A_230_ ratios on a NanoDrop One spectrophotometer (Thermo Fisher Scientific; Waltham, MA, USA), and concentration was measured using the Qubit RNA Assay Kit (Invitrogen, Thermo Fisher Scientific; Waltham, MA, USA) on a Qubit 3.0 Fluorometer (Invitrogen, Thermo Fisher Scientific; Waltham, MA, USA).

### 2.4. miRNA Library Preparation and Sequencing

Due to the lack of previous next generation sequencing miRNome studies in HGSOC, we firstly performed a post-hoc statistical power estimation of the adequate sample size based on the miRNA sequencing results from *n* = 20 samples (*n* = 10 HGSOC tissues and *n* = 10 PCOT) from *n* = 10 patients, with an expected statistical power of 90%. These calculations were performed using the statistical program G*Power version 3.1 (https://www.psychologie.hhu.de/arbeitsgruppen/allgemeine-psychologie-und-arbeitspsychologie/gpower, accessed on 30 June 2021), considering a significance level α of 5%, a power (1-β) of 80%, an effect size (f) of 0.5%, and allocation ratio of 1. Results determine the necessity of a sample size of *n* = 20 per group to reach significant differences between them. Accordingly, the miRNA sequencing experiment was performed in a total cohort of 20 patients, including *n* = 20 HGSOC tissues and *n* = 20 PCOT.

For miRNA sequencing, RNA integrity was investigated using the Agilent RNA 6000 Nano Kit on an Agilent 2100 Bioanalyzer (Agilent Technologies; Santa Clara, CA, USA) and assessed by the RNA integrity number (RIN) [22]. Only those samples with a RIN value of ≥6.0 were considered for miRNA library preparation. 

Library preparation was performed using the CleanTag Small RNA Library Preparation Kit (TriLink Biotechnologies; San Diego, CA, USA) following manufacturer’s protocol. Briefly, 100 ng of the isolated total RNA were employed and sequential steps of 3′ and 5′ adapter ligation, reverse transcription (RT) reaction of tagged RNA library, and PCR amplification were performed from each RNA sample. PCR products were purified following a 2-step protocol with the Agencourt AMPure XP Beads (Beckman Coulter; Brea, CA, USA), yielding a total of 15 μL of purified library. Then, library preparation efficacy was evaluated using the Agilent DNA 1000 Kit on an Agilent 2100 Bioanalyzer (Agilent Technologies; Santa Clara, CA, USA). A 142 pb peak, representing tagged small RNA of ~21 nt in the small RNA libraries, was considered suitable. Finally, miRNA sequencing was performed on an Ion GeneStudio S5 Systems (Ion Torrent, Thermo Fisher Scientific; Waltham, MA, USA), using an Ion 540 Chip (Ion Torrent, Thermo Fisher Scientific; Waltham, MA, USA) with appropriate reagents.

NGS data generated are deposited in the NCBI Gene Expression Omnibus (GEO) under the GEO Series accession number GSE261800 (https://www.ncbi.nlm.nih.gov/geo/query/acc.cgi?acc=GSE261800, accessed on 18 March 2024).

### 2.5. miRNA Sequencing Bioinformatics and Statistical Analyses

All sequencing software tools were run with default or recommended settings under eight cores and 32 GB of RAM computer. The operating system was Ubuntu 20.04.5 LTS using a version of X_86 64 bits.

Read quality and viability was checked using Fastqc software version 0.11.9. Good quality reads were aligned to Homo sapiens miRNAs downloaded from miRBase database (https://mirbase.org/, accesed on 17 February 2022) for acute classification and expression level analysis. Expression level analysis was applied only on miRNAs (mature) displaying a consistent number of sequences/reads (≥150), keeping only those showing a false discovery rate (FDR) correction value < 0.05 after moderated t-statistics by LIMMA (Bioconductor) [23]. Gene set enrichment analysis (GSEA) [24,25] was performed with those significant miRNAs [26], obtaining the biological processes (BP), molecular functions (MF), and cellular components (CC) affected according to the Gene Ontology Consortium (GO). Data are available in the GEO repository (GSE261800). 

### 2.6. miRNA Quantification

For the validation step, a total of *n* = 58 RNA samples was used from HGSOC tissues (*n* = 20), PCOT (*n* = 20), and Caov-3 (*n* = 6), SK-OV-3 (*n* = 6) and SW-626 cells (*n* = 6) in 3 different passages.

cDNA was obtained by RT reaction from 10 ng of total RNA, employing the miRCURY LNA RT Kit (Qiagen; Hilden, Germany), following the manufacturer’s protocol. Afterwards, miRNA expression was analysed by qPCR assays, using the miRCURY SYBR Green Master Mix (Qiagen; Hilden, Germany) as a fluorophore, according to manufacturer’s indications. Briefly, cDNA was 1:60 diluted in RNase-free water and miRCURY SYBR Green Master Mix and specific miRCURY miRNA LNA PCR assays (Qiagen; Hilden, Germany) (Table S1) were added to a 384-well PCR plate in a final reaction volume of 10 μL. qPCR reactions were performed as follows: a polymerase activation/denaturation cycle of 10 min at 95 °C followed by 45 cycles of denaturation for 10 s at 95 °C and 1 min of combined annealing/extension at 56 °C. All RT-qPCR reactions were conducted in a LightCycler 480 II (Roche; Basilea, Switzerland).

The optimal endogenous normaliser was identified from NGS results using NormFinder [27], which ranked candidates by their expression stability in our miRNA sequencing results. A list of *n* = 16.110 combinations of the top stably expressed miRNAs (*n* = 239) and its associated stability values was obtained. The pair of endogenous normalisers were selected according to (1) their expression stability in our experiment, (2) previous experience of the group [28], and (3) their use as endogenous normalisers in the literature [29]. After RT-qPCR analyses, RefFinder [30] was used to evaluate normaliser performance of the selected miRNAs integrating 4 computational algorithms (i.e., Genorm, Delta Ct method, Normfinder, and BestKeeper) and the recommended comprehensive ranking. As a result, we employed the geometric mean of hsa-miR-132-3p and hsa-miR-423-3p for normalisation purposes.

Afterwards, we performed a comprehensive literature review to select the DEmiRNAs of interest to be validated by RT-qPCR. Specifically, the following inclusion criteria were employed: miRNAs reported to be (1) deregulated in cancer; (2) deregulated in OC, and (3) potentially regulated by DNA methylation. In addition to the DEmiRNAs and the pair of endogenous normalisers, the UniSp6 spike-in was included as exogenous control. Relative quantification was determined using the ΔΔCt method [31]. miRNA expression levels were normalized to that of the corresponding control.

### 2.7. DNA Isolation

DNA was extracted from 25 mg of fresh tissues, and from 2·10^5^ OC cells using the DNeasy Blood & Tissue^®^ Kit (Qiagen; Hilden, Germany). The protocol was performed following the manufacturer’s recommendations, with minimal modifications, consisting of an overnight incubation with the provided tissue lysis buffer and the proteinase K of the fresh tissues fragmented with a scalpel and homogenised with the Fisherbrand 150 handheld homogeniser (Fisherbrand, Thermo Fisher Scientific; Waltham, MA, USA).

DNA purity was assessed by quantifying the A_260_/A_280_ and A_260_/A_230_ ratios on a NanoDrop One spectrophotometer (Thermo Fisher Scientific; Waltham, MA, USA), and concentration was measured using the Qubit 1X dsDNA HS Assay Kit (Invitrogen, Thermo Fisher Scientific; Waltham, MA, USA) on a Qubit 3.0 Fluorometer (Invitrogen, Thermo Fisher Scientific; Waltham, MA, USA).

### 2.8. Quantitative DNA Methylation Analysis Using an Illumina 850K BeadChip

DNA methylation levels for miRNAs-associated CpGs of interest were analysed using the Infinium Human MethylationEPIC 850K BeadChip platform (Illumina; San Diego, CA, USA) at the Epigenomics core facility of IIS La Fe (Valencia, Spain) (lead by Dr. Juan Sandoval) on a total cohort of *n* = 8 patients, including *n* = 8 HGSOC tissues and *n* = 8 PCOT. Bisulfite conversion of 600 ng of genomic DNA was performed using the EZ-96 DNA Methylation Kit (Zymo Research; Orange, CA, USA) following the manufacturer’s recommendations. Briefly, samples were whole-genome-amplified followed by an enzymatic endpoint fragmentation, precipitation, and re-suspension. The resuspended samples were hybridised onto the BeadChip for 16 h at 48 °C and washed. A single-nucleotide extension with labelled dideoxynucleotides was performed, and repeated rounds of staining were applied with a combination of labelled antibodies differentiating between biotin and 2,4-dinitrophenol (DNP). Colour balance adjustment and quantile normalisation were performed in order to normalise the samples between the two colour channels. DNA methylation levels were displayed as β-values ranging from 0 to 1. β-values with detection *p* > 0.01 were removed from the analysis. Raw data (IDATs) were normalised and background-corrected using the methylation module (1.9.0) in GENOMESTUDIO (v2011.1) software. Only those Δβ ≥ 0.136 136 (established to differentiate biological relevant methylation changes from background noise and platform variability) [32] and *p*-value < 0.05 were considered for subsequent analyses.

### 2.9. Pyrosequencing Validation of the CpGs of Interest

DNA methylation levels of five CpGs in the proximal promoter (TSS200) of *MIR145* (i.e., cg27083040, cg23917868, cg11671363, cg22941668, and cg08537847) were validated by pyrosequencing. Briefly, 500 ng of genomic DNA were converted using the EZ DNA Methylation Gold (Zymo Research; Orange, CA, USA) bisulfite conversion kit, following the manufacturer’s recommendations. Two specific primer sets (Table S2) for PCR amplification and sequencing were designed (PyroMark assay design v2.0.01.15) to hybridise with CpG-free sites, ensuring methylation-independent amplification. PCR was performed with biotinylated primers, and the PyroMark Vacuum Prep Tool (Biotage; Uppsala, Sweden) was used to prepare single-stranded PCR products. Reactions were performed in a PyroMark Q24 System version 2.0.6 (Qiagen; Hilden, Germany), and methylation values were averaged across the CpG dinucleotides analysed.

### 2.10. Treatment with a Demethylating Agent

For demethylation experiments, 2·10^5^ Caov-3 cells, 10^5^ SK-OV-3 cells and 2·10^5^ SW-626 cells were seeded into 6-well plates and treated with 5-Aza-2′-deoxycytidine (5-Aza) (Sigma-Aldrich, Merck; Darmstadt, Germany) at 0 or 10 μM for 72 h in quadruplicates. 5-Aza supplemented culture media was renewed every 24 h and cells were harvested at 96 h for RNA extraction and RT-qPCR. Results were obtained in three independent experiments.

### 2.11. Cell Lines Transfection

For transfection experiments, 1·10^6^ Caov-3, SK-OV-3, and SW-626 cells were transfected with 50 nM hsa-miR-145-3p mimic (mirVana miRNA mimic) (Ambion, Thermo Fisher Scientific; Waltham, MA, USA) or 20 μM scramble control (BLOCK-iT fluorescent oligo) (Invitrogen, Thermo Fisher Scientific; Waltham, MA, USA) using Lipofectamine RNAiMAX (Invitrogen, Thermo Fisher Scientific; Waltham, MA, USA), according to the manufacturer’s instructions, in a Opti-MEM™ serum-reduced media (Gibco, Thermo Fisher Scientific; Waltham, MA, USA). Then, cells were seeded and used for functional assays at 24 h post-transfection.

### 2.12. Wound Healing Assay

Mimic and scramble control-transfected cells were detached and seeded in 96-well plates in octuplicates. At 95–100% confluence, scratches were generated using the BioTek AutoScratch wound-making tool (Agilent Technologies; Santa Clara, CA, USA). Non-adherent cells were removed by PBS washes. Cells were incubated in their corresponding complete medium for 72 h and pictures were captured every 2 h at the BioTek BioSpa Live Cell Analysis System (Agilent Technologies; Santa Clara, CA, USA) and analysed using the BioTek’s Gen5 analysis software.

### 2.13. Western Blot

Cells were lysed in radioimmunoprecipitation assay buffer containing 20 mM Tris-HCl (pH 8.0), 137 mM NaCl, 1 mM MgCl_2_, 1 mM CaCl_2_, 10% glycerol, 1% NP-40, 0.5% sodium deoxycholate, and 0.1% sodium dodecyl sulfate, along with a protease inhibitor cocktail (#P2714, Sigma-Aldrich, Merck; Darmstadt, Germany). The lysates were incubated at 4 °C for 1 h and then centrifuged at 9300× *g* for 10 min. Western blotting was carried out as described by Pérez-García et al. [33]. The membranes were probed with the following primary antibodies: anti-CCND1 (1:1000, Cell Signaling; Danvers, MA, USA, 92G2 #2978), anti-CDK4 (1:1000, Cell Signaling; Danvers, MA, USA, D9G3E #12790), anti-CDK6 (1:1000, Cell Signaling; Danvers, MA, USA, D4S8S #13331), anti-TUBULIN (1:5000, Abcam; Cambridge, UK, ab6160), and anti-HSP90 (1:5000, Cell Signaling; Danvers, MA, USA, C45G5 #4877). The membranes were then incubated with horseradish peroxidase-conjugated secondary antibodies: anti-rabbit (Bio-Rad; Hercules, CA, USA, 170–6515) and anti-mouse (Bio-Rad; Hercules, CA, USA, 170–6516), each at 1:10,000 dilution. Detection was performed using an enhanced chemiluminescence reaction (GE Healthcare; Chicago, IL, USA, RPN2209) on X-ray films.

### 2.14. Cell Cycle Profile

Mimic and scramble control-transfected cells were detached and seeded in 6-well plates. At approximately 50% confluence, 2·10^5^ cells were fixed with 500 μL of 70% ethanol for at least 1 h at −20 °C. Then, cells were centrifuged, resuspended in 200 μL of propidium iodide (PI) (Sigma-Aldrich, Merck; Darmstadt, Germany) solution at 5 μg/mL with RNAse from bovine pancreas (Sigma-Aldrich, Merck; Darmstadt, Germany) at 100 μg/mL, and incubated overnight at 4 °C. Cell cycle was analysed in Cytoflex 5 flow cytometer (Beckman Coulter; Brea, CA, USA) with the FlowJo software version 10.8.1.

### 2.15. mRNA Quantification

cDNA was obtained by RT reaction from 1 ng of total RNA, employing the NZY First-Strand cDNA Synthesis Flexible Pack (Nzytech; Lisboa, Portugal), following the manufacturer’s protocol. Afterwards, expression was analysed by qPCR assays, using the Vazyme AceQ SYBR^®^ qPCR Master Mix (Vazyme; Nanjing, China), according to manufacturer’s indications. Briefly, cDNA was 1:14 diluted in RNase-free water and Vazyme AceQ SYBR qPCR Master Mix (Vazyme; Nanjing, China) and specific primer pairs (Table S3) were added to a 384-well PCR plate in triplicates in a final reaction volume of 12 μL. qPCR reactions were performed as follows: a polymerase activation/denaturation cycle of 5 min at 95 °C followed by 39 cycles of denaturation for 10 s at 95 °C and 1 min of combined annealing/extension at 60 °C and 72 °C. All RT-qPCR reactions were conducted in a LightCycler 480 II (Roche; Basilea, Switzerland). Relative quantification was determined using the ΔΔCt method [31]. mRNA expression levels were normalized to the expression mean of the controls. The geometric mean of *B2M* and *RPL37A* expression was used as endogenous normalisers, based on literature [34].

### 2.16. TCGA Cohort Analyses

OC data were retrieved from The Cancer Genome Atlas (TCGA) consortium repository (10.1038/nature10166). Clinical and RNA-sequencing information (Illumina HiSeq platform) was downloaded from cBioPortal (https://www.cbioportal.org/study/summary?id=ov_tcga_pub accessed on 10 October 2024).

Survival analyses were performed using univariate Cox regression analysis and Kaplan–Meier (log-rank) test method with clinicopathological variables and dichotomized gene expression levels. Differences between two independent quantitative variables were assessed using Student t test, whereas differences between three independent quantitative variables were assessed using one-way ANOVA. The clinicopathological characteristics of the patients analysed in the TCGA cohort are presented in Table S4.

### 2.17. Statistical Analyses

All variables were checked for normality of the distribution using the Shapiro–Wilk test. Qualitative data descriptions were made using absolute frequencies and percentages. Fisher’s exact test was used to determine association between categorical variables. Quantitative data descriptions were made using the mean ± the standard error of the mean. Differences between two independent quantitative variables were assessed using Student *t* test or Mann–Whitney U test as parametric and non-parametric tests, respectively. Statistical significance levels for the correlations between quantitative variables were calculated using the Spearman’s correlation test. The performance of the diagnostic classification method was analysed using ROC curves. Principal component analyses were also assessed. All statistical tests were considered bilateral and significant with *p*-value < 0.05. Data were analysed using R software (version 3.6.2) (The R foundation, Wien, Austria).

## 3. Results

### 3.1. Deregulation of miRNAs in HGSOC

To gain insight in the epigenetic mechanisms that regulate HGSOC processes, a strategy to identify DEmiRNAs in HGSOC tissues with respect to PCOT was defined (Figure S1A), and high-throughput miRNA sequencing was performed in patient samples (*n* = 40). Sample size was estimated according to appropriate power calculations (Section 2.1. of Material and Methods). The clinico-demographic characteristics of the study cohort are summarized in Table 1. Notably, no statistically significant differences were identified neither in demographic nor in clinical parameters between cohorts under study.

**Figure 1:**
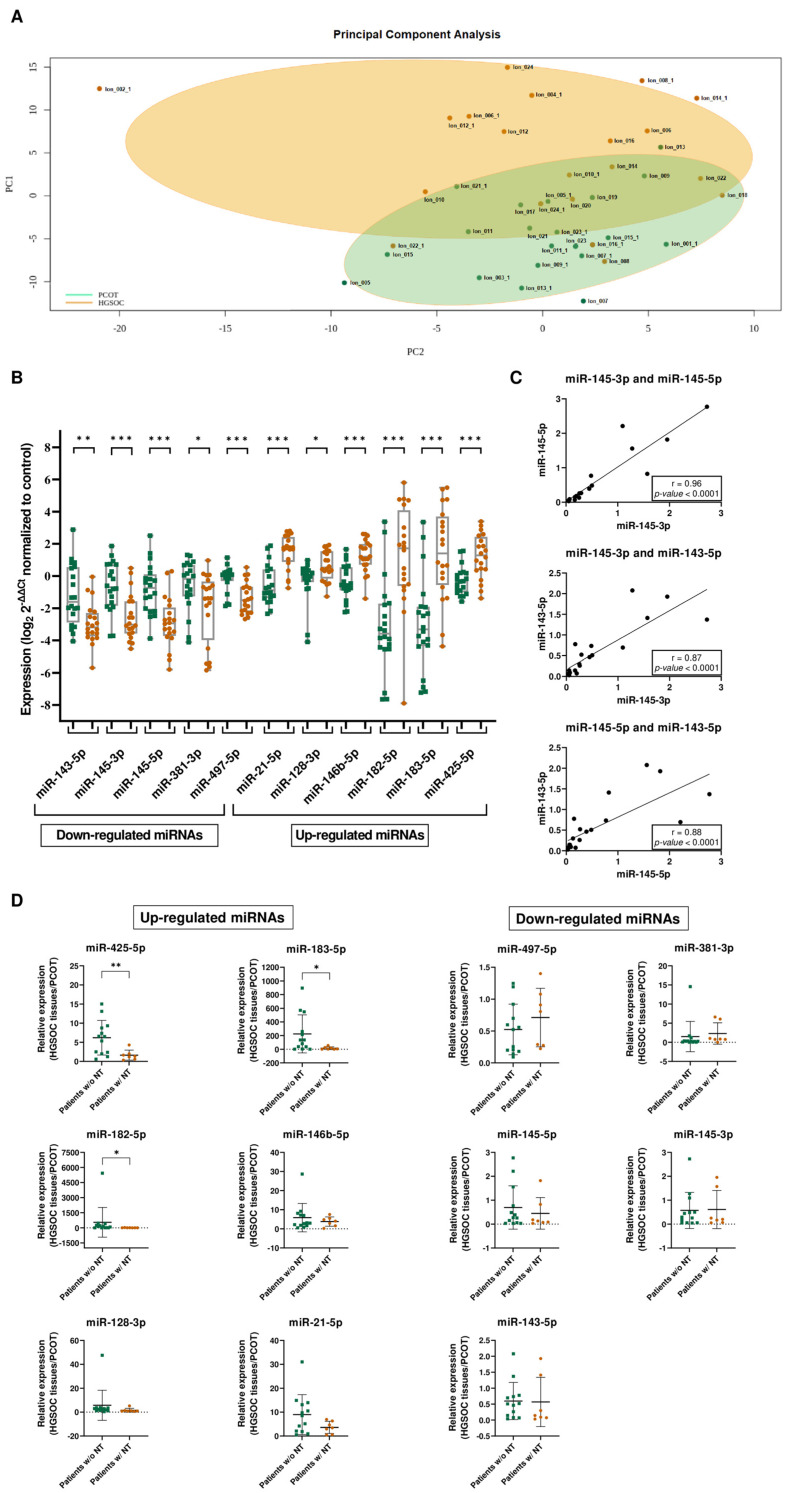
Integrated analysis of miRNA expression profiles in HGSOC: insights from PCA, correlation, and treatment stratification. (**A**) Principal component analysis of miRNA sequencing results assessing the expression of miRNAs between HGSOC and PCOT groups. (**B**) miRNA expression results confirm miRNA sequencing results. RT-qPCR validation of miRNA sequencing results of down- and up-regulated miRNAs in HGSOC tissues (*n* = 20) compared to PCOT (*n* = 20). miRNA expression levels were normalized to the expression levels of their corresponding control. * *p* < 0.05; ** *p* < 0.01; *** *p* < 0.001; and Mann–Whitney U test. (**C**) Correlation between expression levels of miR-145-5p and miR-145-3p, miR-143-5p and miR-145-3p, and miR-143-5p and miR-145-5p. Spearman’s rank correlation. (**D**) Differential expression analysis between patients who received either neoadjuvant treatment (w/NT) or no treatment before surgery (w/o NT) for up-regulated miRNAs and down-regulated miRNAs. NT, neoadjuvant treatment. * *p* < 0.05; ** *p* < 0.01; and Mann–Whitney U test.

Principal component analysis revealed distinct miRNA expression profiles between the HGSOC and PCOT group based on the expression counts on miRNA sequencing experiments (Figure 1A). Subsequent in-depth analysis identified 20 DEmiRNAs, comprising 8 down-regulated and 12 up-regulated miRNAs in HGSOC tissues compared to PCOT (Table S5). Eleven out of the twenty DEmiRNAs were selected for further validation by RT-qPCR in the whole cohort of samples. For normalisation purposes, both miR-132-3p and miR-423-3p were selected as endogenous normalisers, following the previously detailed criteria (materials and methods section) (Figure S1B). RT-qPCR analysis corroborated a significant deregulation of these 11 miRNAs (Figure 1B).

One step further, ROC curve analyses showed the ability of each DEmiRNA to distinguish HGSOC tissues from PCOT, with an area under the curve (AUC) ranging from 0.71 (*p* < 0.05) to 0.84 (*p* < 0.001) for down-regulated miRNAs, and from 0.73 (*p* < 0.05) to 0.88 (*p* < 0.001) for up-regulated miRNAs (Figure S1C). Remarkably, strong correlations were observed between the expression levels of miR-145-5p and miR-145-3p (r = 0.96; *p* < 0.001), and between miR-143-5p and both miR-145 strands: miR-145-3p (r = 0.87; *p* < 0.001) and miR-145-5p (r = 0.88; *p* < 0.001) (Figure 1C). This reinforces the concept of *MIR143HG* as the host gene and a common bicistronic precursor for both pre-miR-143 and pre-miR-145 [35], the latter being the precursor of both miR-145-5p and miR-145-3p.

Considering the effect of chemotherapy, the expression levels of three up-regulated miRNAs (namely miR-425-5p, miR-182-5p, and miR-183-5p) were significantly rescued (*p* < 0.05) in patients with neoadjuvant treatment (NT). Both miR-497-5p and miR-381-3p down-regulated miRNAs showed a trend to recover their expression with the NT, although without reaching statistical significance due to the limited cohort. Interestingly, the levels of miR-143-3p, miR-145-5p, and miR-145-3p were the least affected by the NT, showing minimal changes or no changes between patients who did not receive NT and those who did receive NT (FC: 0.60 vs. 0.57 for miR-145-5p, 0.69 vs. 0.45 for miR-145-3p, and 0.57 vs. 0.61 for miR-143-5p, respectively) (Figure 1D). Remarkably, analyses of the HGSOC cohort (*n* = 389) from the TCGA public available repository indicated an association between increased levels of miR-145-3p and sensitisation to platin salts and taxans-based treatment (Table S4; Figure S2). Altogether, our results reveal a miRNA deregulation in HGSOC and show the ability of the DEmiRNAs to distinguish HGSOC tissues from PCOT. Furthermore, miR-143-3p, miR-145-5p, and miR-145-3p were not sensitive to the neoadjuvant treatment, arising as desirable therapeutic targets.

### 3.2. MIR145 Expression Is Regulated by DNA Methylation in HGSOC Patients

We investigated the contribution of altered DNA methylation to miRNA deregulation in HGSOC. While DNA methylation-mediated deregulation of *MIR145* has been recently described in various cancers [36,37], its role in HGSOC remains unexplored. To this end, we examined over 850,000 methylation sites across the genome by using the Infinium MethylationEPIC 850K BeadChip platform from Illumina. The analysis was conducted on a subset of *n* = 16 paired samples from the miRNA sequencing cohort. Clinico-demographic characteristics of the study cohort are summarized in Table 1.

**Figure 2:**
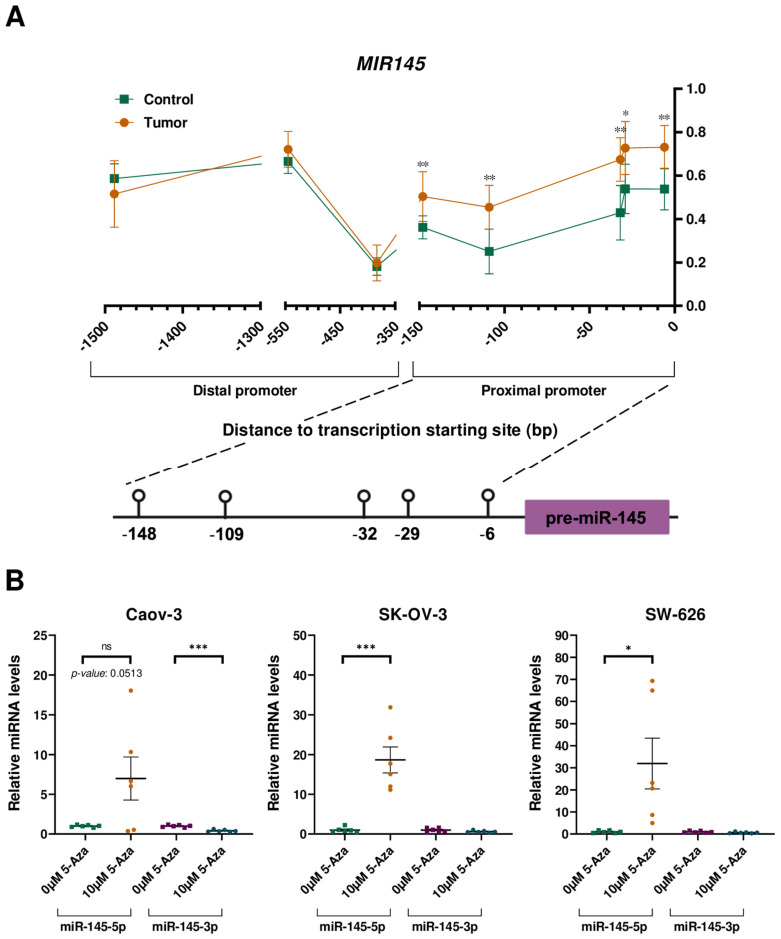
*MIR145* is modulated by DNA methylation. (**A**) Methylation status of the proximal (200 bp upstream of the transcription starting site) and distal (1500 bp upstream of the transcription starting site) promoter of *MIR145*. * *p* < 0.05; ** *p* < 0.01; and Mann–Whitney U test. (**B**) Influence of 5-Aza treatment on the relative expression levels of the miR-145-5p and the miR-145-3p in three OC cell lines. ns: not significant; * *p* < 0.05; *** *p* < 0.001; and Student t-test.

Our analysis focused on 80 CpGs located in the promoter region of the miRNA genes of interest. Among these, four out of eleven miRNA genes exhibited CpGs with differential methylation (∆β ≥ 0.136 and *p*-value < 0.05) between HGSOC tissues and PCOT. Notably, *MIR145* displayed statistically significant differences in methylation levels across five out of eight analysed CpGs (HGSOC tissues vs. PCOT: median ∆β = 0.192; *p* < 0.05). Intriguingly, these five CpGs clustered in a 142bp region in the proximal promoter of the miRNA gene, whereas no statistically significant differences were observed in the three CpGs located at the distal promoter as described previously (Figure 2A). Thus, the hypermethylation status of the CpGs at the proximal promoter of *MIR145* is in agreement with the down-regulated expression of miR-145-5p and miR-145-3p, suggesting the modulation of *MIR145* expression by DNA methylation.

### 3.3. Demethylating Treatment Rescues miR-145-5p but Not miR-145-3p Expression in OC Cell Lines

Then, we examined whether the methylation alterations observed in vivo are recapitulated in vitro. Pyrosequencing investigations revealed that the three OC cell lines (Caov-3, SK-OV-3, and SW-626) presented a similar pattern of *MIR145* hypermethylation compared to patients’ samples (Table S6). These results validate the suitability of these cell lines as a model for studying *MIR145* regulation by DNA methylation in HGSOC.

Treatment of OC cell lines with the demethylating drug 5-Aza restored *MIR145* expression, particularly miR-145-5p, confirming its regulation by DNA methylation (Figure 2B). Strikingly, miR-145-3p mRNA levels were not recovered after treatment with the demethylating agent, suggesting the involvement of an additional repressive mechanism for the expression of this miRNA.

### 3.4. miR-145-3p Overexpression Restrains Tumour Cell Lines Migration

**Figure 3:**
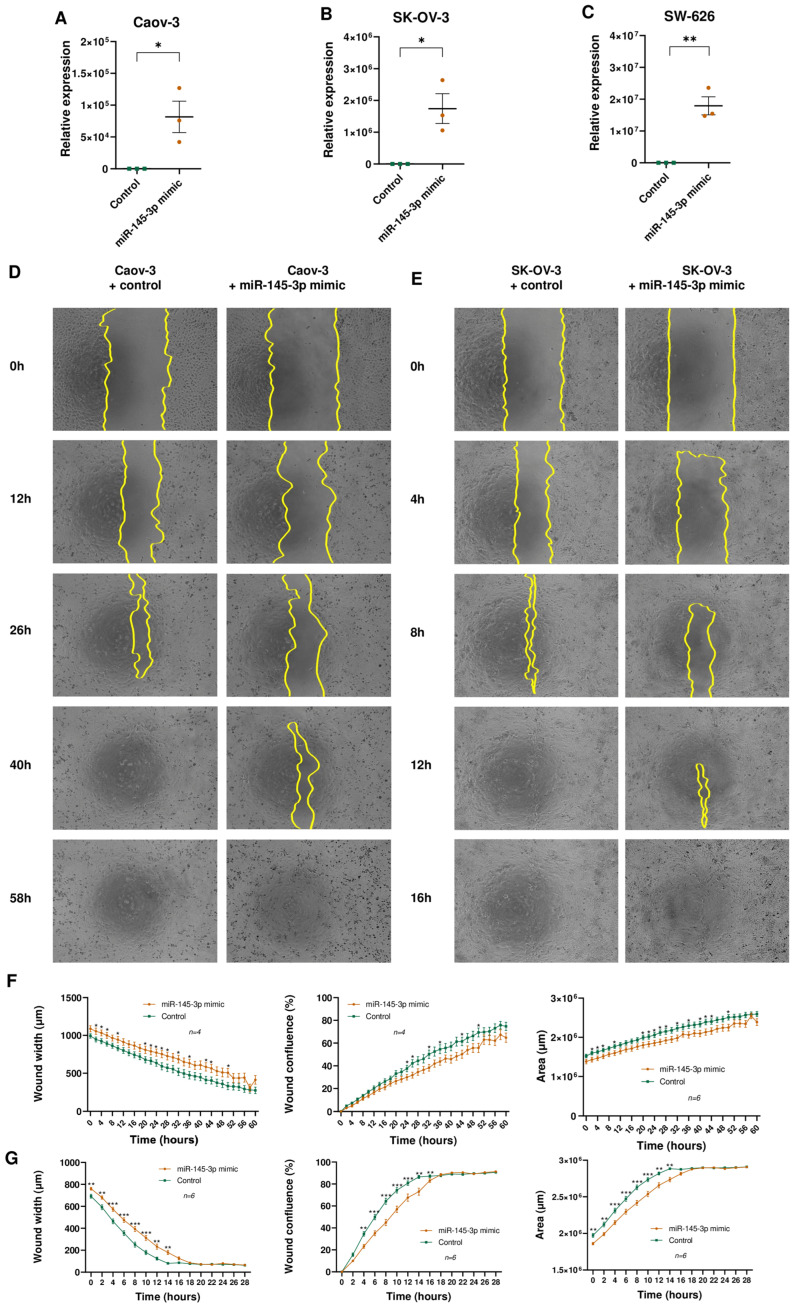
(**A**–**C**) Differential expression analysis of miR-145-3p in (**A**) Caov-3, (**B**) SK-OV-3, and (**C**) SW-626 cells transfected with the miR-145-3p mimic or with a scramble control. * *p* < 0.05; ** *p* < 0.01; and Student t-test. (**D**–**G**) Caov-3 (**D**) and SK-OV-3 (**E**) cells were transfected with the miR-145-3p mimic or with a scramble control; 48 h after transfection, cells were scratch-wounded and were incubated in their appropriate complete medium for 72 h, and pictures were captured every 2 h post-scratching. Yellow lines indicate the wound borders. For Caov-3 (**F**) and SK-OV-3 (**G**): wound confluence (%) represents the fractional area of the wound that is occupied by cells; wound width represents the area of the wound that is not occupied by cells; and the area represents the cell-covered area of the well. * *p* < 0.05; ** *p* < 0.01; *** *p* < 0.001; and Mann–Whitney U test.

Several studies previously implicated miR-145-5p in OC etiology [38,39]. However, the role of miR-145-3p remains relatively unexplored. To address this gap, functional assays were conducted to elucidate the involvement of miR-145-3p in OC pathogenesis. Wound healing assays were performed in OC cell lines (Caov-3, SK-OV-3, and SW-626) to evaluate the impact of miR-145-3p overexpression on tumour cell migration (Figure 3A–C). While SW-626 exhibited comparable cell mobility following transfection with both the miRNA mimic and the control counterpart (Figure S3A–D), overexpression of miR-145-3p led to a significant reduction in cell migration in Caov-3 (Figure 3D,F) and SK-OV-3 (Figure 3E,G) OC cell lines compared to the control group. Subsequent steps involved obtaining miR-145-3p targets predicted by the miRTarBase database (https://mirtarbase.cuhk.edu.cn accessed on 4 February 2023) and performing functional annotation clustering using the Database for Annotation, Visualization, and Integrated Discovery (DAVID) tool (Figure S3E). The clustering analysis revealed a potential regulatory role of miR-145-3p in genes crucial for cell cycle regulation, such as *CCND1*, *CCND2*, and *CDK6* (Figure S3F).

### 3.5. miR-145-3p Overexpression Causes Cell Cycle Arrest at G0/G1 Phase in OC Lines

**Figure 4:**
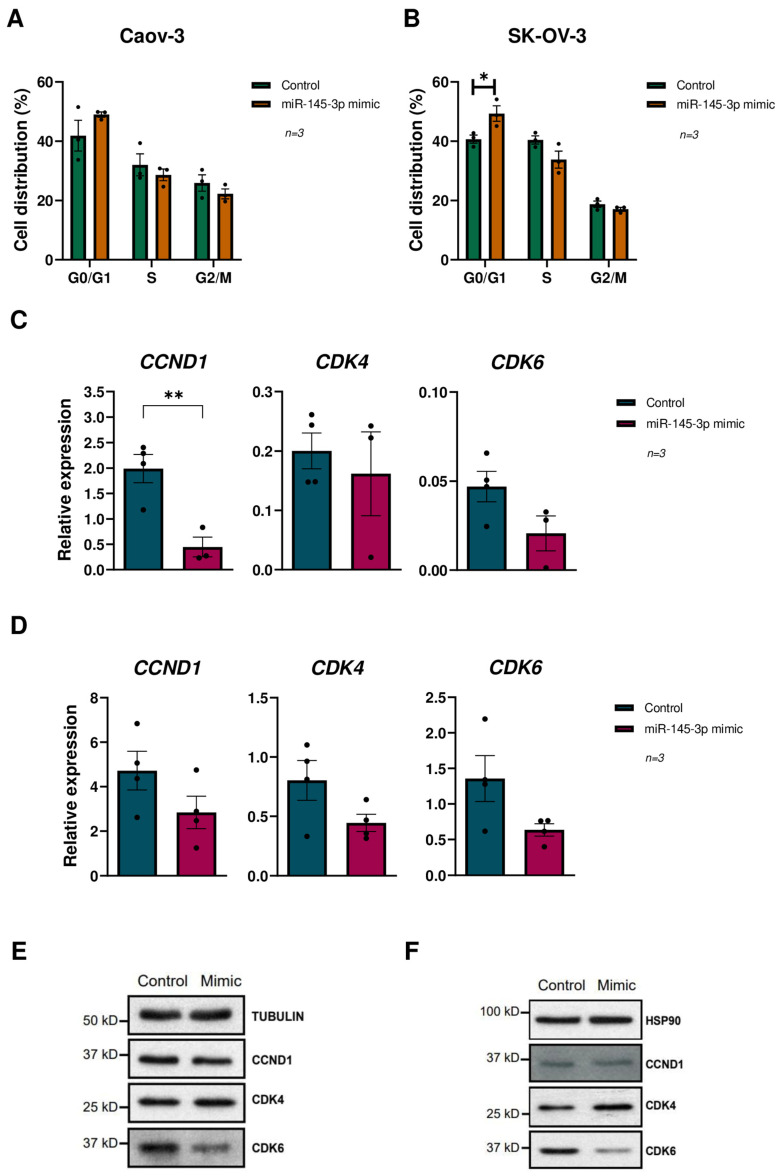
Effects of miR-145-3p on cell cycle and cyclin D1-CDK4/6 pathway regulation in OC cell lines. (**A**,**B**) Cell cycle analysis of: (**A**) Caov-3 and (**B**) SK-OV-3 cells without miR-145-3p expression and cells overexpressing miR-145-3p. * *p* < 0.05; and Student t-test. (**C**,**D**) Differential expression analysis of *CCND1*, *CDK4,* and *CDK6* genes between: (**C**) Caov-3 and (**D**) SK-OV-3 cells without miR-145-3p expression and cells overexpressing miR-145-3p. ** *p* < 0.01; and Student t-test. (**E**,**F**) Western blot analysis of *CCND1*, *CDK4,* and *CDK6* genes between (**E**) Caov-3 and (**F**) SK-OV-3 cells without miR-145-3p expression and cells overexpressing miR-145-3p.

To gain insight into the miR-145-3p’s effect on cell cycle regulation, and to elucidate its underlying molecular mechanism in OC, we investigated its potential involvement in cell proliferation by conducting cell cycle assays in OC cell lines (Caov-3, SK-OV-3, and SW-626) using flow cytometry. Our findings reveal a significant increase in the distribution of cells in the G0/G1 phase upon miR-145-3p overexpression (Figure 4A,B and Figure S4A). These results indicate that miR-145-3p overexpression induces G0/G1 cell cycle arrest in OC cells, consequently leading to reduced cell proliferation.

The cyclin D1-CDK4/6 pathway regulates transition through the G1/S checkpoint of the cell cycle, which is pivotal for cell proliferation [40]. Clustering analysis conducted using miRTarBase-predicted targets of miR-145-3p suggested its potential involvement in the regulation of *CCND1*, *CCND2*, and *CDK6*. Consequently, we assessed the mRNA expression of *CCND1*, *CCND2*, *CDK4*, and *CDK6* via RT-qPCR following miR-145-3p overexpression in OC cells, resulting in a reduced mRNA expression of *CCND1*, *CDK4*, and *CDK6* in all cell lines (Figure 4C,D and Figure S4B), while *CCND2* was undetectable. Further analysis at the protein levels showed that whereas the overexpression of miR-145-3p did not affect *CCND1* or *CDK4* in any of the 3 OC lines, *CDK6* levels were reduced in the Caov-3 and SK-OV-3 OC lines (Figure 4E,F), whereas no changes were observed in SW-626 cells (Figure S4C). Overall, our findings reveal the downregulation of miR-145-3p in HGSOC and demonstrate reduced cell migration and G0/G1 arrest in OC cell lines overexpressing miR-145-3p. This suggests that miR-145-3p plays a role in regulating cell cycle regulation through the modulation of the cyclin D1-CDK4/6 pathway (Figure S3F).

## 4. Discussion

OC is one of the most prevalent gynecologic malignancies, with approximately 95% of cases arising from epithelial cells [41,42]. Within the spectrum of epithelial ovarian cancers (EOC), HGSOC emerges as the most frequent and aggressive epithelial ovarian cancer subtype, presenting a 5-year OS rate of around 50%, coupled with limited therapeutic options [2,43]. The pressing need to unravel the molecular intricacies driving HGSOC aggressiveness, particularly through epigenomic approaches, prompted the present study to scrutinize the miRNA signature of HGSOC, explore its potential regulation by DNA methylation, and delve into the functional implications of miR-145-3p in cell proliferation.

The application of high-throughput miRNA sequencing in this study yielded a comprehensive miRNA signature, highlighting DEmiRNAs between HGSOC ovarian tissue and corresponding non-cancerous ovarian tissue (PCOT). This signature positions miRNA sequencing as a robust tool for identifying DEmiRNAs in HGSOC overcoming probe-based miRNA arrays largely employed in previous discovery studies [44,45,46]. Comparison with previous microarray studies and other sequencing approaches not only validated consistency, but also introduced novelty to the landscape of HGSOC-related DEmiRNAs. Noteworthy is the scale of this study, encompassing the largest cohort to date [47,48], including 20 pairs of HGSOC and PCOT, making it a pioneering effort in comparing miRNA expression profiles through high-throughput miRNA sequencing. Strikingly, the validated DEmiRNAs clearly distinguished HGSOC tissues from PCOT, suggesting their potential as minimally invasive biomarkers and paving the way for future investigations into their diagnostic and prognostic utility.

Focused attention on *MIR145*, housing down-regulated miRNAs in HGSOC tissues (miR-145-5p and miR-145-3p), unraveled a downregulation of both strands, accentuating their potential as antitumour miRNAs. Furthermore, DNA methylation analysis uncovered deregulated CpGs in the promoter region of several miRNA genes, with prominent alterations in *MIR145*’s proximal promoter, highlighting the convergence of DNA methylation and miRNA dysregulation in HGSOC, offering valuable insights into potential therapeutic targets. Remarkably, apart from our data on DNA demethylating agents, a growing body of evidence demonstrates that some natural compounds and drugs (i.e., curcumin or sodium butyrate) increase the expression of a myriad of miRNAs, including miR-145-5p, miR-143, and/or miR-21 [49,50,51,52].

The study’s unique contribution lies in the examination of the functional impact of miR-145-3p on cell migration and cell cycle regulation. The findings open novel dimensions in understanding HGSOC pathogenesis, providing evidence that miR-145-3p plays a crucial role in inhibiting cell proliferation and inducing G0/G1 phase arrest in HGSOC cells matching with the regulation of its target gene *CCND1* and *CDK6*. While miR-145-5p downregulation [38,39] and its role in *CDK6* regulation [36] have been extensively reported in HGSOC, the study introduces the novel aspect of miR-145-3p, shedding light on its functional significance in the disease-controlling cell proliferation through the cyclin D1-CDK4/6 pathway, with potential therapeutic implications.

One step further, the analyses performed on the HGSOC TCGA cohort provided insights in the potential involvement of miR-145-3p in the sensitisation of HGSOC to platin salts and taxanes-based treatments (Figure S2). Considering gynaecological cancers, miR-145-5p over-expression has been reportedly involved in sensitization of cervical cancer cells to mitomycin [53] and radiotherapy [54,55]. Particularly in ovarian cancer, several miRNAs (e.g., miR-142-5p, miR-181c, and miR-1271) modulate the sensitivity of tumoural cells to cisplatin and/or paclitaxel-based therapies, mainly through modulation of the PI3K/Akt/mTOR axis [56,57,58]. Therefore, the observed association between increased levels of miR-145-3p and sensitization to chemotherapy might be explained by the fact that several up-stream regulators (e.g., IGF1R, EGFR, and KRAS) and downstream effectors (e.g., cyclin D1, cyclin D2, and *CDK6*) of the PI3K/Akt/mTOR axis are encountered among miR-145-3p targets.

Considering the recent advances in the ovarian cancer’s therapeutic arsenal, CDK4/6 inhibitors are emerging as valuable agents in the setting of different solid malignances, both alone or in combination with other drugs, in agreement with their success in estrogen receptor-positive metastatic breast cancer treatment [59]. Promising preclinical results [60] fueled a myriad of ongoing Phase 1/2 clinical trials (i.e., NCT04553133, NCT06188520, NCT06307249, NCT06552858, NCT06243185, NCT04315233, NCT04469764, NCT06264921, NCT05768139, NCT05238922, NCT03519178, and NCT02465060) [61]. Strikingly, the DNA damage repair machinery impairment provoked by CDK4/6 inhibitors might synergistically act with the increased genomic instability rendered by poly ADP-ribose polymerase inhibitors [62]. In this context, concomitant miR-145-3p-mediated downregulation of *CDK6* might be considered in the oncoming strategy in HGSOC therapy.

Despite the significant strides made in this study, certain limitations, such as the non-rescue of miR-145-3p after demethylating treatment, are acknowledged. However, the research presented here marks a substantial advancement in unraveling the intricate molecular landscape of HGSOC, leveraging high-throughput sequencing and DNA methylation arrays. The identification and validation of DEmiRNAs, particularly the exploration of miR-145-3p, significantly enriches the understanding of HGSOC biology, offering potential avenues for therapeutic intervention. The study’s strength lies in its robust methodology, including a sizable cohort and the integration of cutting-edge epigenomic techniques. This work not only contributes to the expanding knowledge of HGSOC, but also lays the foundation for future investigations into the clinical implications of the identified miRNA signature and the therapeutic potential of miR-145-3p in HGSOC.

## Figures and Tables

**Table 1:** Clinico-demographic characteristics of patients of study.

Study Population	HGSOC Patients (*n* = 20 Patients; *n* = 40 Tissues)	HGSOC Patients (*n* = 8 Patients; *n* = 16 Tissues)	*p*-Value
Assay	miRNA Sequencing	EPIC 850K DNA Methylation Array	
Age (years) (mean ± SEM)	60.10 ± 2.70	61.50 ± 3.99	ns ^a^
BMI (kg/m^2^) (mean ± SEM)	24.89 ± 1.02	26.44 ± 1.71	ns ^a^
Ethnicity (n (%))			ns ^b^
Caucasian (Spanish)	18 (90.00)	8 (100.00)	
Caucasian (European non-Spanish)	2 (10.00)	0 (0.00)	
FIGO Stage (n (%))			ns ^b^
I–II	7 (35.00)	5 (62.50)	
III–IV	13 (65.00)	3 (37.50)	
Neoadjuvant treatment (n (%))			ns ^b^
Yes	7 (35.00)	0 (0.00)	
No	13 (65.00)	8 (100.00)	
PCI (mean ± SEM)	8.50 ± 1.51	9.38 ± 3.03	ns ^a^

BMI, body mass index; FIGO, international federation of gynecology and obstetrics; HGSOC, high-grade serous ovarian cancer; ns, not significant; PCI, peritoneal carcinomatosis index; and SEM, standard error of the mean. ^a^ Student *t*-test; ^b^ Fisher’s exact test.

## Data Availability

NGS data generated throughout this work have been deposited in the NCBI Gene Expression Omnibus (GEO) and can be retrieved using the GEO Series accession number GSE261800 (https://www.ncbi.nlm.nih.gov/geo/query/acc.cgi?acc=GSE261800 accessed on 18 March 2024). All other data used and/or analysed during the current study are available from the corresponding author on reasonable request.

## Supplementary Materials

The following supporting information can be downloaded at: https://www.mdpi.com/article/10.3390/cells13221904/s1, Table S1. ID of the sets of primers (miRCURY miRNA LNA PCR assays (Qiagen; Hilden, Germany)) for RT-qPCR reactions for miRNA quantification of 11 DEmiRNAs, the pair of endogenous normalizers (hsa-miR-132-3p and hsa-miR-423-3p) and the UniSp6 spike-in included as exogenous control. Table S2. Sets of primers for PCR amplification and pyrosequencing validation of five CpGs in the proximal promoter (TSS200) of *MIR145*. Table S3. Sets of primers for RT-qPCR reactions for mRNA quantification of genes involved in cell cycle (*CCND1*, *CCND2*, *CDK4*, *CDK6*) and the housekeeping genes used for normalization (*B2M*, *RPL37A*). Table S4. Clinicopathological characteristics of patients of the TCGA cohort with available miRNA-seq data. Table S5. miRNA sequencing experiment reveals DEmiRNAs (n = 20) in the final cohort. Down-regulated miRNAs (n = 8) are in the left column and the up-regulated ones (n = 12) in the right column. Table S6. Methylation level of the 5 statistically significant differentially methylated CpGs (DMCpGs) in the proximal promoter of the *MIR145* gene measured by the Infinium Methyla-tionEPIC 850K BeadChip in HGSOC tissues and by pyrosequencing in OC cell lines. Figure S1. (A) Schematic representation of the study design. (B) miR-132-3p and miR-423-3p are the best endogenous normalizers in our cohort. Analysis of the miRNA stability from qRT-PCR results in our samples by the RefFinder comprehensive tool. Each graph represents the 14 miRNAs and the geometric mean of miR-132-3p and miR-423-3p by each algorithm: Genorm, Delta Ct method, NormFinder, Comprehensive ranking, and BestKeeper. The lower the stability value, the higher the stability of each miRNA. (C) DEmiRNAs distinguish HGSOC tissues from PCOT. ROC curves obtained for up-regulated miRNAs and down-regulated miRNAs. AUC: area under the ROC curve; CI: confidence interval. Figure S2. Distribution of miR-145-3p and miR-145-5p levels in patients of the TCGA co-hort considering: (A, C) Complete response to chemotherapy/radiotherapy treatment vs no re-sponders. (B, D) Patients sensitive to platinum-based treatment vs resistant patients. * *p* < 0.05; ns, not significant. Figure S3. miR-145-3p regulates cell cycle in OC cell lines via three potential putative pre-dicted targets. (A) Effect of miR-145-3p transfection on Caov-3 cells migration in vitro: cells were transfected with the miR-145-3p mimic or with a control. 48h after transfection, cells were scratch-wounded and were incubated in their appropriate complete medium for 72 hours, and pictures were captured every 2 hours post-scratching. Yellow lines indicate the wound borders. (B) Wound width represents the area of the wound that is not occupied by cells. ** *p* < 0.01; *** *p* < 0.001; Mann-Whitney U test. (C) Wound confluence (%) represents the fractional area of the wound that is occupied by cells. ** *p* < 0.01; *** *p* < 0.001; Mann-Whitney U test. (D) The area represents the cell-covered area of the well. ** *p* < 0.01; *** *p* < 0.001; Mann-Whitney U test. (E) Schematic repre-sentation of the steps taken to identify 3 putative miR-145-3p targets: *CCND1*, *CCND2* and *CDK6*. (F) *CDK4/6* regulates the transition from the G1 phase to the S phase of the cell cycle. The transition through the G1/S checkpoint is regulated by the cyclin D1-*CDK4/6* pathway, which commits a cell to proliferation. Cyclin D1 binds to dimerized *CDK4*/6 and phosphorylates and inactivates pRb, resulting in the release of E2F transcription factors and the activation of genes involved in cell proliferation. Our hypothesis is that miR-145-3p reduces cell proliferation by targeting *CCND1*, *CCND2* and *CDK6*. Figure S4. Effects of miR-145-3p on cell cycle and cyclin D1-CDK4/6 pathway regulation in SW-626 cell line. (A) Cell cycle analysis of SW-626 cells without miR-145-3p expression and cells overexpressing miR-145-3p. Student t-test. (B) Differential expression analysis of CCND1, CDK4 and CDK6 genes between SW-626 cells without miR-145-3p expression and cells overexpressing miR-145-3p. Student t-test. (C) Western Blot analysis of CCND1, CDK4 and CDK6 genes between SW-626 cells without miR-145-3p expression and cells overexpressing miR-145-3p.

## Abbreviations

5-Aza	5-Aza-2′-deoxycytidine
AUC	Area under the curve
BMI	Body mass index
BP	Biological processes
CC	Cellular components
DAVID	Database for Annotation, Visualization, and Integrated Discovery
DEmiRNA	Differentially expressed miRNA
FDR	False discovery rate
FIGO	International Federation of Gynecology and Obstetrics
GEO	Gene expression omnibus
GO	Gene ontology
GSEA	Gene set enrichment analysis
HGSOC	High-grade serous ovarian cancer
MF	Molecular functions
NGS	Next generation sequencing
NT	Neoadjuvant treatment
OC	Ovarian cancer
OS	Overall survival
PARPi	Poly(ADP-Pibose)polymerase inhibitors
PCI	Peritoneal carcinomatosis index
PCOT	Paired control non-tumoural ovarian tissue
PI	Propidium iodide
RIN	RNA integrity number
ROC	Receiver operating characteristic
TCGA	The Cancer Genome Atlas
