# Supplementary material for: Reduced Levels of miR-145-3p Drive Cell Cycle Progression in Advanced High-Grade Serous Ovarian Cancer
Source: Cells. 2024 Nov 18;13(22):1904. doi: 10.3390/cells13221904 (PMC11592657; doi:10.3390/cells13221904)
Supplement: Supplementary file 1 [file cells-13-01904-s001.zip › cells-3233048-supplementary.pdf]

cells

## 5. Supplementary Material

### 5.1. Tables

**Table S1.** ID of the sets of primers (miRCURY miRNA LNA PCR assays (Qiagen; Hilden, Germany)) for RT-qPCR reactions for miRNA quantification of 11 DEmiRNAs, the pair of endogenous normalizers (hsa-miR-132-3p and hsa-miR-423-3p) and the UniSp6 spike-in included as exogenous control.

| miRNA | ID |
|---|---|
| hsa-miR-425-5p | ID 204337 |
| hsa-miR-183-5p | ID 206030 |
| hsa-miR-182-5p | ID 206070 |
| hsa-miR-146b-5p | ID 2119310 |
| hsa-miR-128-3p | ID 205995 |
| hsa-miR-21-5p | ID 204230 |
| hsa-miR-497-5p | ID 204354 |
| hsa-miR-381-3p | ID 205887 |
| hsa-miR-145-5p | ID 204483 |
| hsa-miR-145-3p | ID 204192 |
| hsa-miR-143-5p | ID 204570 |
| hsa-miR-132-3p | ID 206035 |
| hsa-miR-423-3p | ID 204488 |
| UniSp6 | ID 203954 |

**Table S2.** Sets of primers for PCR amplification and pyrosequencing validation of five CpGs in the proximal promoter (TSS200) of *MIR145*.

| Region | CpG | Forward primer | Reverse primer |
|---|---|---|---|
| Region 1 | cg27083040 | 5'-TTGGTAGGAGATTGGGGAATA-3' | 5'-[btn]CCTAAAAACCAACTAAAATTCTCTTCTACA-3' |
| | cg23917868 | | |
| Region 2 | cg11671363 | 5'-GGGTTGGATGTAGAAGAGAATT-3' | 5'-[btn]TTCCAAAAATCCCCATCTTAACAT-3' |
| | cg22941668 | | |
| | cg08537847 | | |

**Table S3.** Sets of primers for RT-qPCR reactions for mRNA quantification of genes involved in cell cycle (*CCND1, CCND2, CDK4, CDK6*) and the housekeeping genes used for normalization (*B2M, RPL37A).*

| Gene | Forward primer | Reverse primer |
|---|---|---|
| ***CCND1*** | 5’-TCTACACCGACAACTCCATCCG-3’ | 5’-TCTGGCATTTTGGAGAGGAAGTG-3’ |
| ***CCND2*** | 5’-GAGAAGCTGTCTCTGATCCGCA-3’ | 5’-CTTCCAGTTGCGATCATCGACG-3’ |
| ***CDK4*** | 5’-CCATCAGCACAGTTCGTGAGGT-3’ | 5’-TCAGTTCGGGATGTGGCACAGA-3’ |
| ***CDK6*** | 5’-GGATAAAGTTCCAGAGCCTGGAG-3’ | 5’-GCGATGCACTACTCGGTGTGAA-3’ |
| ***B2M*** | 5’-CCACTGAAAAAGATGAGTATGCCT-3’ | 5’-CCAATCCAAATGCGGCATCTTCA-3’ |
| ***RPL37A*** | 5’-AATCAGCCAGCACGCCAAGTAC-3’ | 5’-GCCACTGTCTTCATGCAGGAAC-3’ |

*CCND1*, cyclin D1; *CCND2*, cyclin D2; *CDK4*, cyclin-dependent kinase 4; *CDK6*, cyclin-dependent kinase 6; *B2M*, beta-2-microglobulin; *RPL37A*, ribosomal protein L37a

**Table S4.** Clinicopathological characteristics of patients of the TCGA cohort with available miRNA-seq data.

| Characteristics | N (389) | % |
|---|---|---|
| **Age at surgery (years) (mean, range)** | 60 (30-87) | |
| **Race** | | |
| White | 340 | 87.4 |
| Black | 21 | 5.4 |
| Asian | 13 | 3.3 |
| Unknown | 15 | 3.9 |
| **FIGO staging** | | |
| II | 19 | 4.9 |
| III | 310 | 79.7 |
| IV | 60 | 15.4 |
| **Exitus** | | |
| No | 140 | 36.0 |
| Yes | 249 | 64.0 |
| **Treatment response** | | |
| Complete | 81 | 20.8 |
| Progression | 262 | 67.4 |
| Unknown | 46 | 11.8 |
| **Platinum response** | | |
| Sensitive | 152 | 39.1 |
| Resistant | 63 | 16.2 |
| Unknown | 174 | 44.7 |

**Table S5. miRNA sequencing experiment reveals DEmiRNAs (n=20) in the final cohort.** Down-regulated miRNAs (n=8) are in the left column and the up-regulated ones (n=12) in the right column.

| DEREGULATED miRNAs (n=20) | | | | | |
|---|---|---|---|---|---|
| DOWN-REGULATED miRNAs (n=8) | | | UP-REGULATED miRNAs (n=12) | | |
| ID | Fold-change | Adjusted *p-value* | ID | Fold-change | Adjusted *p-value* |
| hsa-miR-1-3p | -0.47 | <0.01 | hsa-miR-183-5p | 1.64 | <0.01 |
| hsa-miR-195-3p | -0.43 | <0.001 | hsa-miR-182-5p | 0.68 | <0.001 |
| hsa-miR-145-5p | -0.41 | <0.001 | hsa-miR-142-5p | 0.37 | <0.001 |
| hsa-miR-299-3p | -0.41 | <0.001 | hsa-miR-1307-3p | 0.34 | <0.001 |
| hsa-miR-143-5p | -0.30 | <0.001 | hsa-miR-210-3p | 0.31 | <0.01 |
| hsa-miR-145-3p | -0.28 | <0.01 | hsa-miR-155-5p | 0.31 | <0.001 |
| hsa-miR-497-5p | -0.24 | <0.001 | hsa-miR-425-5p | 0.27 | <0.001 |
| hsa-miR-381-3p | -0.20 | <0.01 | hsa-miR-425-3p | 0.27 | <0.01 |
| | | | hsa-miR-146b-5p | 0.22 | <0.001 |
| | | | hsa-miR-21-5p | 0.20 | <0.001 |
| | | | hsa-miR-222-3p | 0.14 | <0.001 |
| | | | hsa-miR-128-3p | 0.13 | <0.001 |

**Table S6.** Methylation level of the 5 statistically significant differentially methylated CpGs (DMCpGs) in the proximal promoter of the *MIR145* gene measured by the Infinium MethylationEPIC 850K BeadChip in HGSOC tissues and by pyrosequencing in OC cell lines.

| ID | β-value average (% ± SD) | | | | |
|---|---|---|---|---|---|
| | Infinium MethylationEPIC 850K BeadChip | | Pyrosequencing | | |
| | PCOT | HGSOC tissues | Caov-3 | SK-OV-3 | SW-626 |
| **cg27083040** | 36.16 ± 13.09 | 50.35 ± 11.41 | 95.36 ± 3.11 | 92.17 ± 0.91 | 71.55 ± 2.79 |
| **cg23917868** | 25.08 ± 14.14 | 45.42 ± 10.12 | 69.50 ± 1.55 | 62.74 ± 2.38 | 8.17 ± 1.15 |
| **cg11671363** | 42.91 ± 15.30 | 67.39 ± 10.06 | 95.36 ± 0.16 | 72.56 ± 4.93 | 97.29 ± 0.01 |
| **cg22941668** | 53.88 ± 15.53 | 72.69 ± 12.24 | 92.53 ± 1.44 | 96.40 ± 0.62 | 97.16 ± 0.04 |
| **cg08537847** | 53.83 ± 13.53 | 73.06 ± 9.96 | 87.70 ± 1.61 | 88.72 ± 3.72 | 89.93 ± 2.55 |

SD, standard deviation; PCOT, paired control ovarian tissue; HGSOC, high-grade serous ovarian cancer.

## *5.2. Figures*

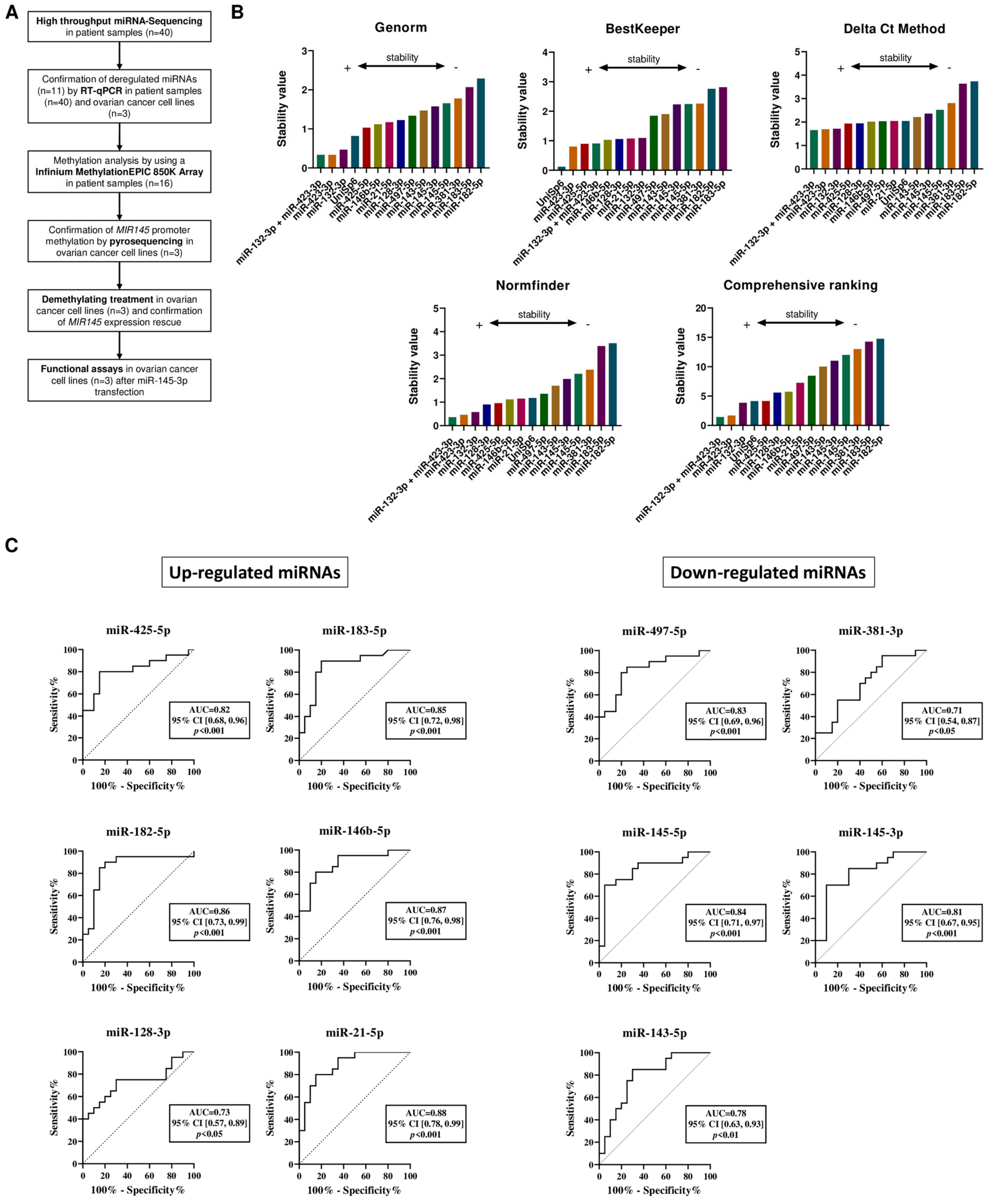


**Figure S1. (A)** Schematic representation of the study design. **(B)** miR-132-3p and miR-423-3p are the best endogenous normalizers in our cohort. Analysis of the miRNA stability from qRT-PCR results in our samples by the RefFinder comprehensive tool. Each graph represents the 14 miRNAs and the geometric mean of miR-132-3p and miR-423-3p by each algorithm: Genorm, Delta Ct method, NormFinder, Comprehensive ranking, and BestKeeper. The lower the stability value, the higher the

stability of each miRNA. **(C)** DEmiRNAs distinguish HGSOC tissues from PCOT. ROC curves obtained for up-regulated miRNAs and down-regulated miRNAs. AUC: area under the ROC curve; CI: confidence interval.

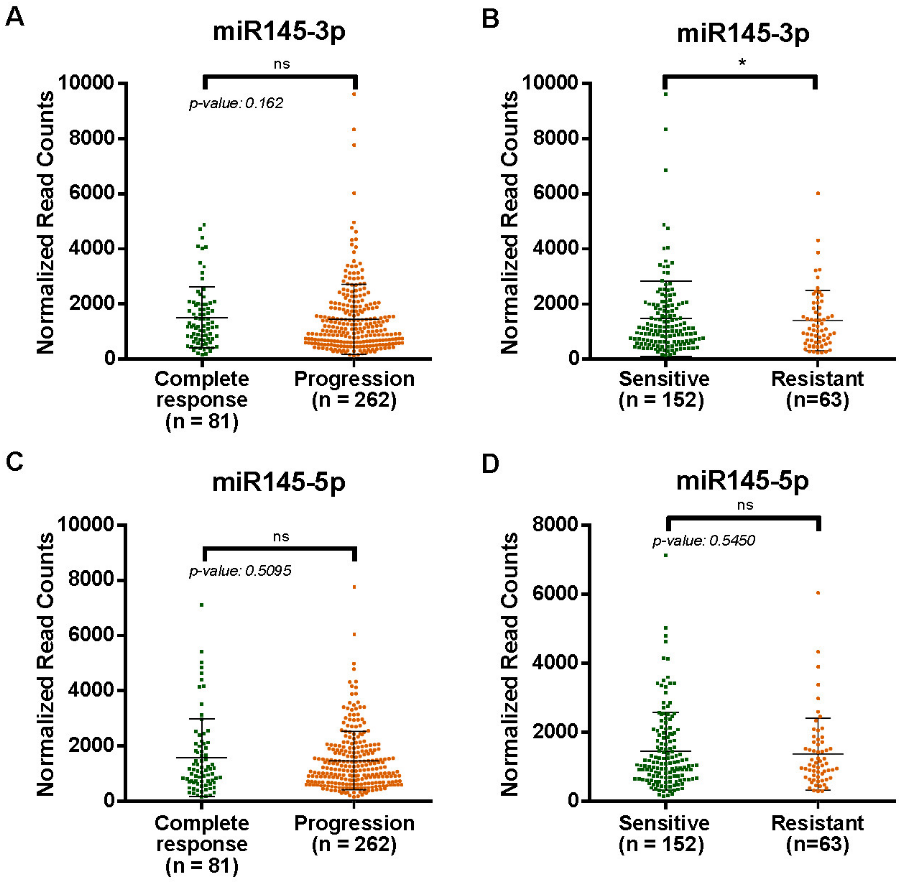


**Figure S2. Distribution of miR-145-3p and miR-145-5p levels in patients of the TCGA cohort considering: (A, C)** Complete response to chemotherapy/radiotherapy treatment vs no responders. **(B, D)** Patients sensitive to platinum-based treatment vs resistant patients. * $p<0.05$; ns, not significant

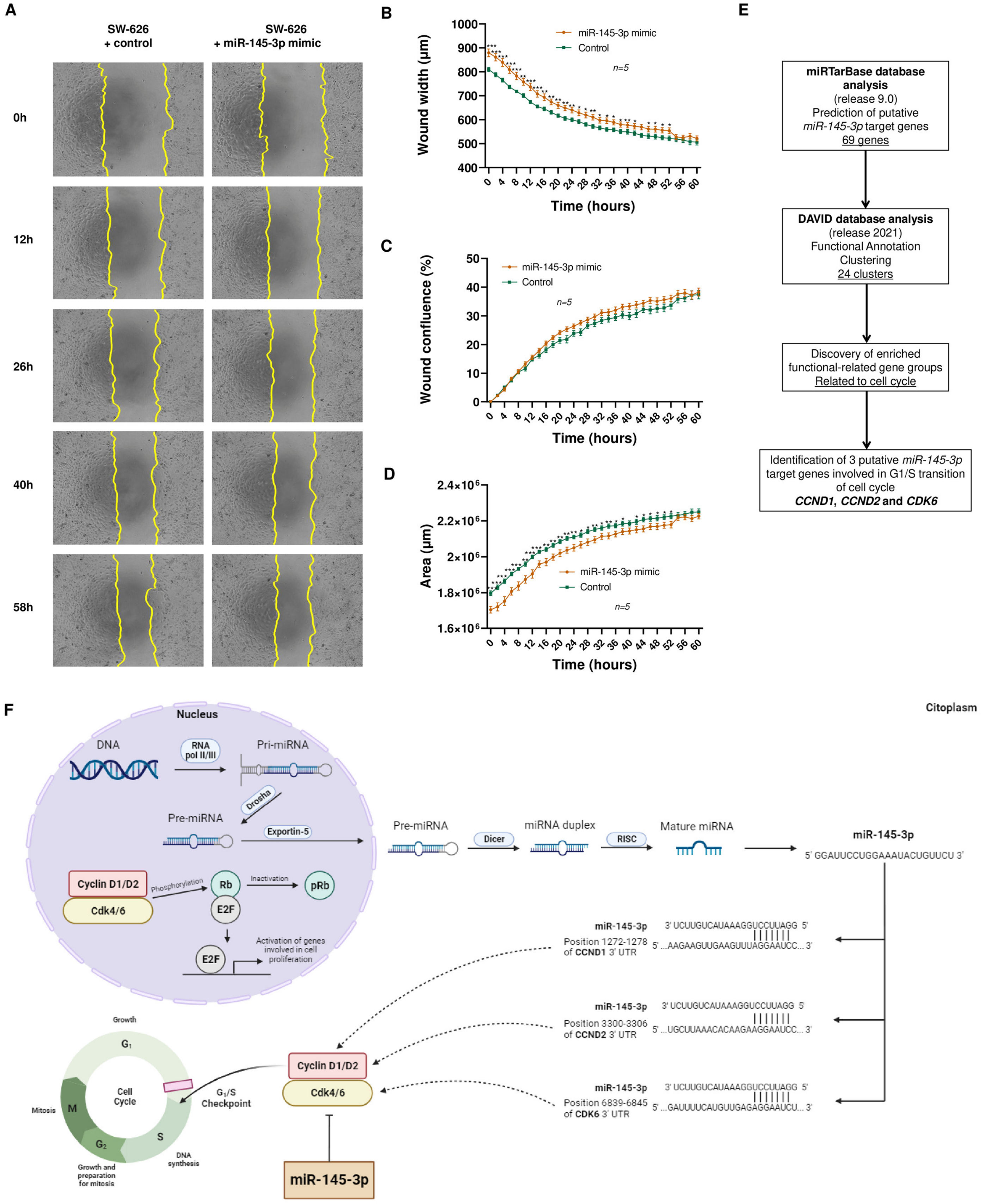


**Figure S3. miR-145-3p regulates cell cycle in OC cell lines via three potential putative predicted targets. (A)** Effect of miR-145-3p transfection on Caov-3 cells migration in vitro: cells were transfected with the miR-145-3p mimic or with a control. 48h after transfection, cells were scratch-wounded and were incubated in their appropriate complete medium for 72 hours, and pictures

were captured every 2 hours post-scratching. Yellow lines indicate the wound borders. **(B)** Wound width represents the area of the wound that is not occupied by cells. ** p<0.01; *** p<0.001; Mann-Whitney U test. **(C)** Wound confluence (%) represents the fractional area of the wound that is occupied by cells. ** p<0.01; *** p<0.001; Mann-Whitney U test. **(D)** The area represents the cell-covered area of the well. ** p<0.01; *** p<0.001; Mann-Whitney U test. **(E)** Schematic representation of the steps taken to identify 3 putative miR-145-3p targets: *CCND1*, *CCND2* and *CDK6*. **(F)** *CDK4/6* regulates the transition from the G1 phase to the S phase of the cell cycle. The transition through the G1/S checkpoint is regulated by the cyclin D1-*CDK4/6* pathway, which commits a cell to proliferation. Cyclin D1 binds to dimerized *CDK4/6* and phosphorylates and inactivates pRb, resulting in the release of E2F transcription factors and the activation of genes involved in cell proliferation. Our hypothesis is that miR-145-3p reduces cell proliferation by targeting *CCND1*, *CCND2* and *CDK6*.

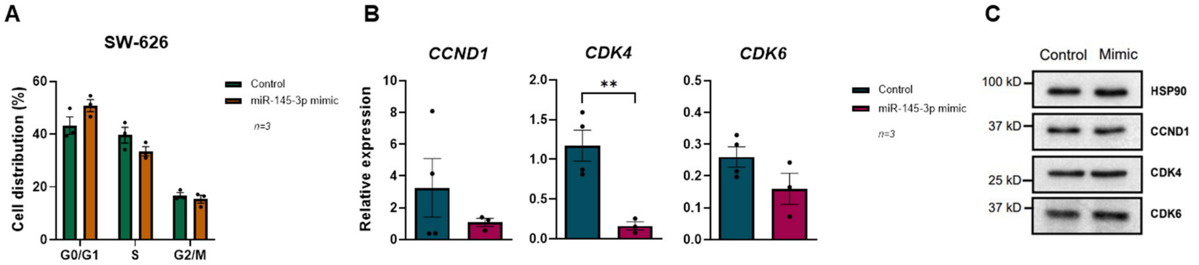


**Figure S4. Effects of miR-145-3p on cell cycle and cyclin D1-CDK4/6 pathway regulation in SW-626 cell line. (A)** Cell cycle analysis of SW-626 cells without miR-145-3p expression and cells overexpressing miR-145-3p. Student t-test. **(B)** Differential expression analysis of CCND1, CDK4 and CDK6 genes between SW-626 cells without miR-145-3p expression and cells overexpressing miR-145-3p. Student t-test. **(C)** Western Blot analysis of CCND1, CDK4 and CDK6 genes between SW-626 cells without miR-145-3p expression and cells overexpressing miR-145-3p.

**Disclaimer/Publisher's Note:** The statements, opinions and data contained in all publications are solely those of the individual author(s) and contributor(s) and not of MDPI and/or the editor(s). MDPI and/or the editor(s) disclaim responsibility for any injury to people or property resulting from any ideas, methods, instructions or products referred to in the content.
